# The Effectors and Sensory Sites of Formaldehyde-responsive Regulator FrmR and Metal-sensing Variant * [Fn FN2]

**DOI:** 10.1074/jbc.M116.745174

**Published:** 2016-07-29

**Authors:** Deenah Osman, Cecilia Piergentili, Junjun Chen, Lucy N. Sayer, Isabel Usón, Thomas G. Huggins, Nigel J. Robinson, Ehmke Pohl

**Affiliations:** From the ‡ Department of Chemistry,; § School of Biological and Biomedical Sciences, Durham University, Durham DH1 3LE, United Kingdom,; ¶ Procter and Gamble, Mason Business Center, Cincinnati, Ohio 45040,; the ‖ Instituto de Biología Molecular de Barcelona (IBMB-CSIC), Barcelona Science Park, 08028 Barcelona, Spain, and; the ** Institució Catalana de Recerca i Estudis Avançats (ICREA), Pg. Lluís Companys 23, 08010 Barcelona, Spain

**Keywords:** allosteric regulation, Escherichia coli (E. coli), metal, Salmonella enterica, zinc, RcnR/CsoR, cobalt, formaldehyde, glutathione

## Abstract

The DUF156 family of DNA-binding transcriptional regulators includes metal sensors that respond to cobalt and/or nickel (RcnR, InrS) or copper (CsoR) plus CstR, which responds to persulfide, and formaldehyde-responsive FrmR. Unexpectedly, the allosteric mechanism of FrmR from *Salmonella enterica* serovar Typhimurium is triggered by metals *in vitro*, and variant FrmR^E64H^ gains responsiveness to Zn(II) and cobalt *in vivo*. Here we establish that the allosteric mechanism of FrmR is triggered directly by formaldehyde *in vitro*. Sensitivity to formaldehyde requires a cysteine (Cys^35^ in FrmR) conserved in all DUF156 proteins. A crystal structure of metal- and formaldehyde-sensing FrmR^E64H^ reveals that an FrmR-specific amino-terminal Pro^2^ is proximal to Cys^35^, and these residues form the deduced formaldehyde-sensing site. Evidence is presented that implies that residues spatially close to the conserved cysteine tune the sensitivities of DUF156 proteins above or below critical thresholds for different effectors, generating the semblance of specificity within cells. Relative to FrmR, RcnR is less responsive to formaldehyde *in vitro*, and RcnR does not sense formaldehyde *in vivo*, but reciprocal mutations FrmR^P2S^ and RcnR^S2P^, respectively, impair and enhance formaldehyde reactivity *in vitro*. Formaldehyde detoxification by FrmA requires *S*-(hydroxymethyl)glutathione, yet glutathione inhibits formaldehyde detection by FrmR *in vivo* and *in vitro*. Quantifying the number of FrmR molecules per cell and modeling formaldehyde modification as a function of [formaldehyde] demonstrates that FrmR reactivity is optimized such that FrmR is modified and *frmRA* is derepressed at lower [formaldehyde] than required to generate *S*-(hydroxymethyl)glutathione. Expression of FrmA is thereby coordinated with the accumulation of its substrate.

## Introduction

Formaldehyde (H_2_C=O), as a strong electrophile, is capable of alkylating and cross-linking the reactive groups (such as thiols and amines) of proteins and DNA (1–5). This reactivity and subsequent damage to biological macromolecules make formaldehyde a highly cytotoxic compound. In addition to environmental sources, formaldehyde is generated intracellularly by a number of cellular processes. In methylotrophic and methanotrophic bacteria, it is well known that formaldehyde is generated as a by-product of methanol and methane oxidation (6–9), consistent with the presence of genetically encoded formaldehyde detoxification systems in these organisms (2, 8, 10–12). Intracellular formaldehyde generation in bacteria that do not use these C_1_ substrates as a carbon source has been less well studied. Formaldehyde is produced by the alternative heme degradation pathway (IsdG and IsdI) in *Staphylococcus aureus* to acquire iron (13, 14). The recent detection of trimethylamine *N*-oxide (TMAO) ^3^ demethylase activity in cell extracts suggests that this activity may be an endogenous source of formaldehyde in *Escherichia coli* (15). Demethylation of nucleic acids and production of methylglyoxal from glyceraldehyde 3-phosphate and dihydroxyacetone phosphate during glycolysis represent more widespread physiological sources of formaldehyde (16–18). In addition, several mechanisms for the generation of formaldehyde at the host-pathogen interface have recently been proposed (2).

**FIGURE 1.**
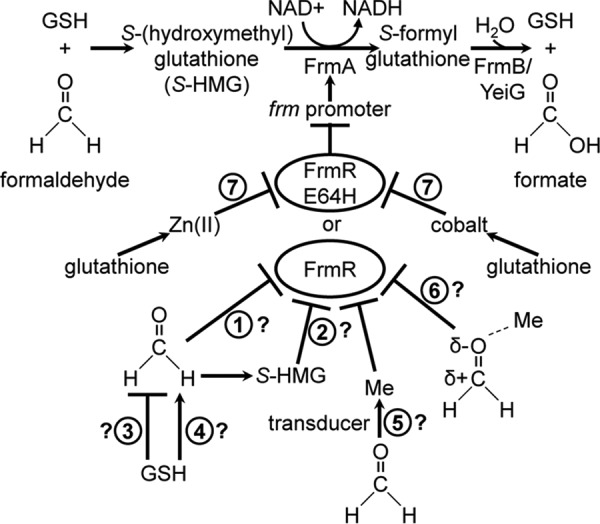
**Formaldehyde detoxification and sensing.** Spontaneous reaction of formaldehyde with GSH generates *S*-(hydroxymethyl)glutathione (*S-HMG*), the substrate oxidized by FrmA to *S*-formylglutathione (28). Following hydrolysis of *S*-formylglutathione by FrmB, formate is produced as the final product, and GSH is regenerated (22, 29). An additional enzyme, YeiG, is also implicated in formaldehyde detoxification because it demonstrates hydrolytic activity against *S*-formylglutathione, although *yeiG* is not FrmR-regulated (27). Notably, FrmB is present in the *E. coli* but not the *Salmonella frm* operon. *Salmonella* FrmR (or FrmR^E64H^) represses expression from the *frm* promoter, which is alleviated by exogenous formaldehyde. The intracellular effector of (any) FrmR is unknown, and possibilities include formaldehyde alone (*1*) or *S*-(hydroxymethyl)glutathione (*2*), in which case GSH could act negatively (*3*) or positively (*4*) on FrmR-mediated derepression. Alternatively, FrmR derepression may be transduced by a metal intermediate (*5*) or require activation of formaldehyde by metal (*6*). FrmR^E64H^ additionally responds to Zn(II) and cobalt; however, the response to metals is lost in cells lacking glutathione (*7*) (50).

**FIGURE 2.**
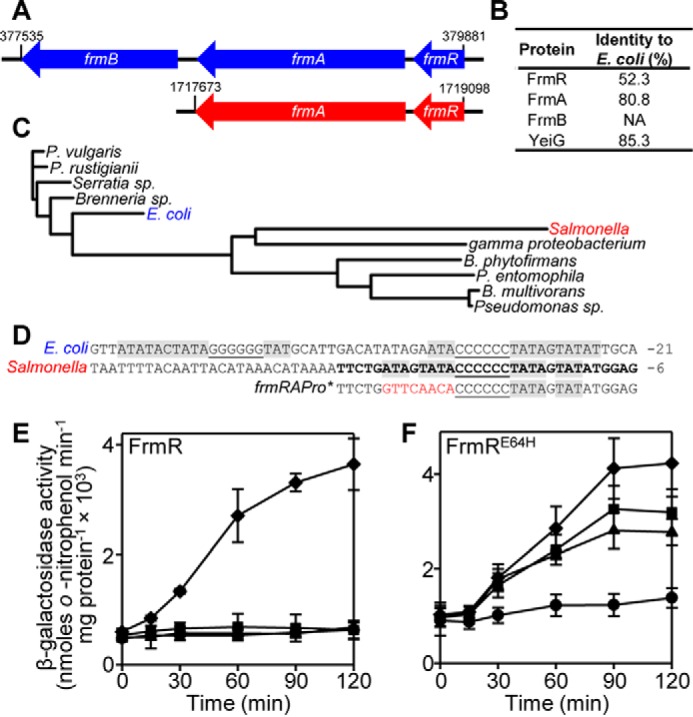
***E. coli* and *Salmonella* FrmRs have distinct origins but *Salmonella* FrmR and FrmR^E64H^ retain their effector sensitivities in *E. coli*.** *A*, schematic representation of the *frm* operon (to scale) from *E. coli* K12 (*blue*) and *Salmonella* (strain SL1344; *red*), with the nucleotide position at the start and end of each gene cluster indicated. *B*, percentage identity of *Salmonella* proteins required for formaldehyde detoxification compared with their *E. coli* orthologue. *C*, rooted phylogenetic tree of 11 sequences from the DUF156 FrmR subgroup identified previously (45). Organism details and UniProtKB identifiers are outlined under “Experimental Procedures.” *D*, alignment of the *frm* promoter from *E. coli* and *Salmonella.* The position relative to the translational start site is *labeled*. G/C tracts are *underlined*. T/A-rich inverted repeats are highlighted in *gray*. The sequence corresponding to one strand of *frmRA*Pro, used for fluorescence anisotropy, is in *boldface type*. Mutations to generate *frmRA*Pro* are highlighted in *red. E* and *F*, β-galactosidase activity as a function of time in *E. coli* BW25113Δ*frmR* containing P*_frmRA_-frmR* (*E*) or P*_frmRA_-frmR^E64H^* (*F*), fused to *lacZ* following exposure of logarithmic cells to MNIC formaldehyde (50 μm; *diamonds*), Zn(II) (50 μm; *triangles*), Co(II) (5 μm; *squares*), or untreated control (*circles*). Values are means of at least three biological replicates (each performed in triplicate) with S.D. (*error bars*).

Inducible formaldehyde detoxification mechanisms have now been recognized in most bacteria (2, 3, 19). A glutathione-dependent pathway represents the most widespread formaldehyde detoxification system, although the functional proteins and/or genomic arrangement may vary (19–26). In *E. coli*, this pathway is encoded by the *frmRAB* operon, which includes *frmA*, encoding a Zn(II)-binding glutathione-dependent formaldehyde dehydrogenase, and *frmB*, encoding *S*-formylglutathione hydrolase (Figs. 1 and 2*A*) (26–29). Regulation of the *frmRAB* operon upon formaldehyde accumulation is mediated by the first gene product, FrmR, a DNA-binding transcriptional regulator (26).

FrmR is a member of the RcnR/CsoR family (DUF156) of (predominantly) metal-sensing transcriptional repressors (30–32). This family can be divided into subgroups that have evolved to detect distinct and specific effectors in a cellular context by modification of a relatively conserved protein scaffold, in a manner similar to ArsR, MerR, and Fur family regulators (33–36). In addition to FrmR, characterized DUF156 subgroups to date include the metal sensors RcnR and DmeR, which respond to Ni(II)/Co(II); CsoR and RicR, which respond to Cu(I); InrS, which responds to Ni(II); and the non-metal sensor CstR, which undergoes cysteine modification by sodium sulfite, selenite, and tellurite (31, 32, 37–41). Upon binding of an allosteric effector (*e.g.* metal ion), affinity for DNA is weakened, alleviating repression from the target operator-promoter (30). At the time of writing, CsoR represents the only member of this family for which a structure has been reported (31, 42–44). CsoR forms a three-helix bundle that adopts a tetrameric assembly made up of a dimer of dimers. The known effector sensory sites of metal-sensing DUF156 proteins exploit side chains of conserved residues at a dimer interface, denoted the *WXYZ* fingerprint, characteristic of each subgroup but all involving a conserved Cys-thiolate (position *X*) located at the amino-terminal end of helix α2 (31, 45, 46). Analogous information is not yet available for the sensory sites of FrmR.

*E. coli* FrmR-mediated transcriptional repression is alleviated following exposure of cells to exogenous formaldehyde, CO-releasing molecules, and chloride treatment and during anaerobic respiration using TMAO as the terminal electron acceptor (15, 26, 47–49). However, the effector directly detected by FrmR in each case remains unexplored. We recently identified an FrmR homologue in *Salmonella enterica* serovar Typhimurium strain SL1344 (hereafter referred to as *Salmonella*), which, as observed for *E. coli*, responds to exogenous formaldehyde *in vivo* (Fig. 1) (50). Unlike *E. coli* FrmR (containing two), *Salmonella* FrmR possesses three (four including Glu^64^) putative metal ligands at positions *WXY*, within the metal-binding fingerprint of metal-sensing DUF156 members (50). Moreover, *Salmonella* FrmR can bind Co(II), Cu(I), and Zn(II). Unexpectedly, Cu(I) and Zn(II) are capable of triggering an allosteric response that weakens FrmR DNA affinity *in vitro* (50). Metal responsiveness is not observed *in vivo* because FrmR is less sensitive than the endogenous *Salmonella* sensors for these metals. However, generation of a variant FrmR, responsive to cobalt and Zn(II) in addition to formaldehyde *in vivo*, is achieved by single amino acid substitution at the putative metal-binding site (Glu^64^ → His) (Fig. 1). The combined effect of tighter metal affinity and weaker DNA affinity of the apo-form, relative to wild type FrmR, confers metal-sensing gain of function to FrmR^E64H^ (50). Evidence that *Salmonella* FrmR is competent to respond to metals raises the possibility that formaldehyde sensing could be indirect and mediated by an effect on metal availability to FrmR by formaldehyde (Fig. 1). Notably, FrmA also requires Zn(II) for catalytic activity (47). The extent to which Zn(II) might be required to act as a signal transducer of formaldehyde accumulation in a cell now needs to be addressed.

In addition to FrmR, transcriptional regulators that respond following exposure to exogenous formaldehyde include HxlR (MarR family) from *Bacillus subtilis* and NmlR/AdhR (MerR family) identified in *Neisseria* sp. and other Gram-positive pathogens (51–56). However, the effector(s) detected by any formaldehyde-responsive transcriptional regulator has yet to be biochemically identified. Despite the requirement of glutathione for formaldehyde detoxification by FrmA, the extent to which glutathione plays a role in the regulation of expression of glutathione-dependent formaldehyde dehydrogenase in any organism is unknown (Fig. 1).

We present the first *in vitro* evidence that formaldehyde is a direct allosteric effector of *Salmonella* FrmR. The FrmR sensory site is particularly reactive to formaldehyde such that the related *Salmonella* RcnR sensor is less responsive to formaldehyde *in vitro* and *in vivo*. We determine the crystal structure of FrmR^E64H^ to define the effectors and sensory site(s) of this formaldehyde- and metal-sensing variant. Residues required for Zn(II)/Co(II) and formaldehyde sensing are determined and support a mechanism involving an FrmR-specific formaldehyde cross-link between Pro^2^ and Cys^35^. An RcnR variant with enhanced sensitivity for formaldehyde *in vitro* was generated based on the deduced FrmR sensory site and mechanism. Implications for the basis of effector specificity within DUF156 family proteins and the chemical species detected by FrmR *in vivo* are discussed.

## Results

### 

#### 

##### Salmonella FrmR and FrmR^E64H^ Retain Responsiveness to Formaldehyde and Metals When Expressed in E. coli

Despite *E. coli* and *Salmonella* being co-linear for most genes (57), the *Salmonella frm* operon occurs at a distinct genomic location compared with *E. coli* and lacks the *frmB* gene for *S*-formylglutathione hydrolase (Fig. 2*A*). *E. coli* and *Salmonella* FrmRs share only 52.3% sequence identity (Fig. 2*B*) compared with an average ∼85% identity for orthologous gene products between these organisms (58, 59), and analysis of the DUF156 FrmR subgroup demonstrates that they are polyphyletic (Fig. 2*C*). This is also reflected by the distinct operator-promoter sequences upstream of each *frm* locus (Fig. 2*D*). The significance of these differences in relation to formaldehyde detoxification remains unknown but may reflect specific requirements for formaldehyde detoxification in the respective cellular backgrounds. To investigate the response of *Salmonella* FrmR in an *E. coli* cytosol, P*_frmRA_-frmR* reporter constructs comprising the *Salmonella frmRA* promoter (P*_frmRA_*) and *frmR* coding sequence fused to *lacZ* were expressed in *E. coli* cells that lacked the endogenous *E. coli frmR* gene (Δ*frmR*) (Fig. 2*E*). As observed in *Salmonella* (50), expression from P*_frmRA_-frmR* was derepressed in the heterologous *E. coli* host following exposure of cells to maximum non-inhibitory concentrations (MNICs) of formaldehyde, whereas exposure to MNIC CoCl_2_ and ZnCl_2_ did not alleviate repression (Fig. 2*E*). FrmR variant, FrmR^E64H^, which responds to CoCl_2_, ZnCl_2_, and formaldehyde in *Salmonella* cells (50), retains the same effector responsiveness when P*_frmRA_-frmR^E64H^* is expressed in *E. coli* Δ*frmR* (Fig. 2*F*). This demonstrates that the ability of FrmR^E64H^ to respond to metals (and formaldehyde) *in vivo* is not exclusive to *Salmonella* cells.

##### FrmR Senses Formaldehyde Directly

**FIGURE 3.**
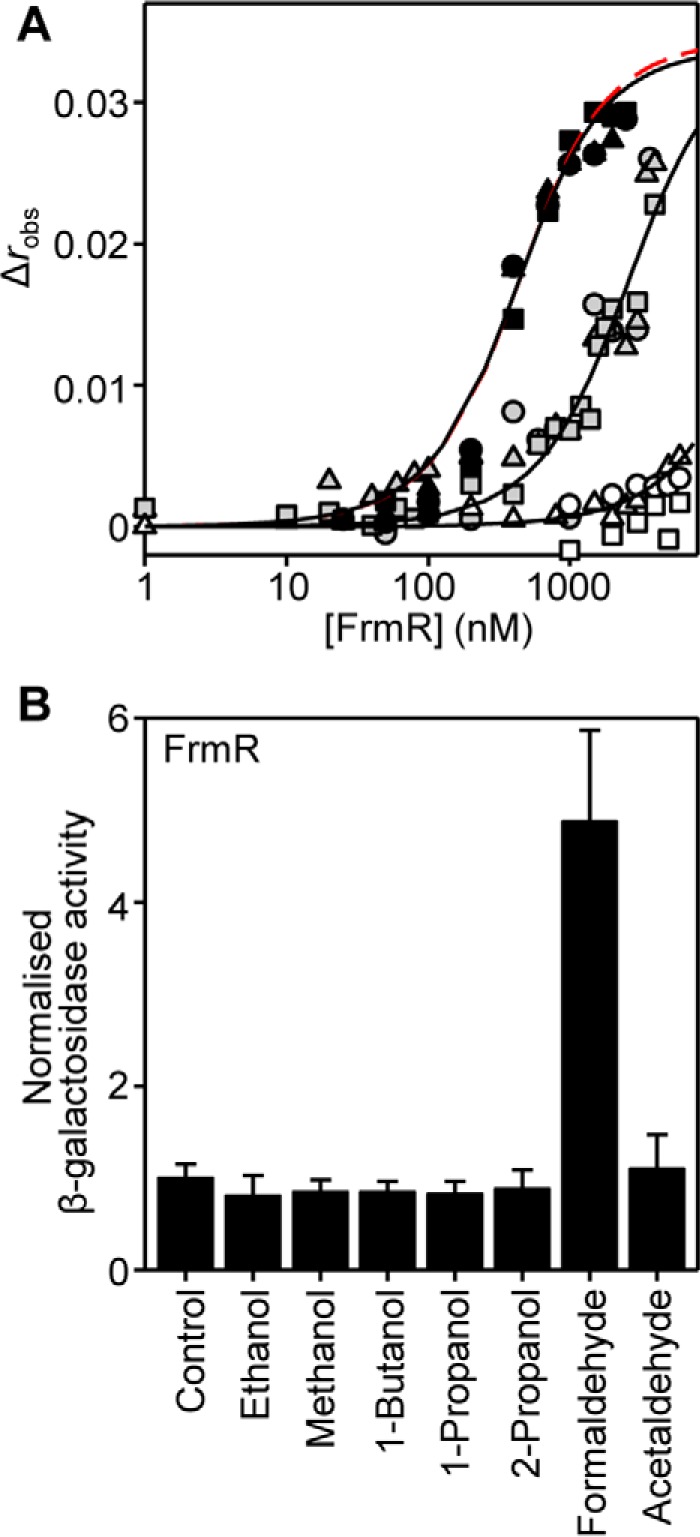
**FrmR responds specifically to formaldehyde *in vitro* and *in vivo*.** *A*, anisotropy change upon titration of a limiting concentration of *frmRA*Pro (10 nm) with FrmR in the presence of 5 mm EDTA and either 20 μm acetaldehyde (*black symbols*), 10 μm formaldehyde (*gray symbols*), or 20 μm formaldehyde (*open symbols*). *Symbol shapes* represent individual experiments. Data were fit to a model describing a 2:1 protein tetramer (nondissociable)/DNA stoichiometry (binding with equal affinity) (50, 86), and *lines* represent simulated curves produced from the average (apparent) *K*_DNA_ determined across the experimental replicates shown. The *dashed red line* (*largely obscured*) is a simulated curve based on the published *K*_DNA_ of apo-FrmR (50), presented here for comparative purposes. *B*, β-galactosidase activity in SL1344 containing P*_frmRA_-frmR* fused to *lacZ* grown to mid-exponential phase in M9 minimal medium in the absence (control) or presence of MNIC indicated alcohol or aldehyde (see “Experimental Procedures” for concentrations). Values are means of three biological replicates (each performed in triplicate) with S.D. (*error bars*).

**FIGURE 4.**
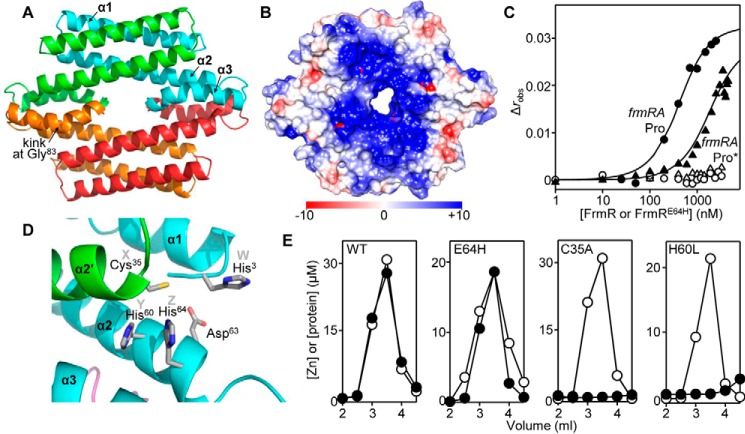
**Structure of FrmR^E64H^ and inferred Zn(II)/Co(II)-sensing site.** *A*, *ribbon representation* of the 2.19 Å resolution crystal structure of FrmR^E64H^ tetramer (Protein Data Bank code 5LCY; see Table 2 for a summary of the crystallographic data). Each monomer is *colored* differently, and secondary structural units are *labeled* on the *cyan* monomer. *B*, electrostatic surface potential of FrmR^E64H^ tetramer using Chimera (103). The *color scale* is from −10 (negative potential; *red*) to +10 (positive potential; *blue*) kcal/mol·e. *C*, anisotropy change upon titration of a limiting concentration (10 nm) of *frmRA*Pro (*solid symbols*) or *frmRA*Pro* (half-site defined in Fig. 2*D*; *open symbols*) with FrmR (*circles*) or FrmR^E64H^ (*triangles*) in the presence of 5 mm EDTA. The *lines* are fits of the data to a model describing a 2:1 protein tetramer (nondissociable)/DNA stoichiometry (binding with equal affinity) (50, 86). *D*, expansion of the dimeric interface with backbone helices from two different monomers *shaded green* and *cyan* (the *same colors* as used in *A).* The inferred Zn(II)/Co(II)-binding site comprises Cys^35^ from α2′, and His^60^ and His^64^ from α2 (belonging to the *XYZ* motif required for metal binding in DUF156 members CsoR, RcnR, and InrS (39, 46, 68), with His^3^ from α1 (position *W* (46, 61)) and Asp^63^ presenting candidate fourth ligands. *E*, analysis of fractions (0.5 ml) for protein by Bradford assay (*open circles*) and metal by inductively coupled plasma MS (*filled circles*) following size exclusion chromatography of FrmR, FrmR^E64H^, FrmR^C35A^ (50 μm, monomer), or FrmR^H60L^ (in this case, [monomer] = 32.5 μm), preincubated with 150 μm ZnCl_2_.

Repression by FrmR (and FrmR^E64H^) is alleviated by exogenous formaldehyde *in vivo* (Fig. 2, *E* and *F*), but DNA binding to the target *frmRA* operator-promoter (*frmRA*Pro) (Fig. 2*D*) is weakened by Zn(II) (and Cu(I)) *in vitro* (50). To explore whether the *in vivo* response might be transduced by metals during formaldehyde stress or whether formaldehyde is able to act directly on FrmR, fluorescence anisotropy was used to monitor the interaction of FrmR with fluorescently labeled *frmRA*Pro in the presence of formaldehyde (Fig. 3*A*). FrmR has previously been shown to bind *frmRA*Pro with a stoichiometry of two tetramers per DNA molecule and a *K*_DNA_ of 9.9 ± 0.3 × 10^−8^ m for each tetramer, in the absence of effector (50) (also confirmed here in Fig. 4*C*). Consequently, a limiting concentration (10 nm) of *frmRA*Pro was used for titration with FrmR in the presence of 10 or 20 μm formaldehyde, concentrations chosen to minimize nonspecific formaldehyde cross-linking, which is likely at higher formaldehyde concentrations (60). EDTA was included as a metal chelator to eliminate any effect that may arise due to the presence of (allosterically effective) trace metals. The anisotropy data were fit to a model describing the binding of two non-dissociable FrmR tetramers per DNA molecule and revealed that DNA binding of FrmR to *frmRA*Pro was weakened by ∼6.5-fold and ≥70-fold (compared with the published value (50); Table 1) in the presence of 10 and 20 μm formaldehyde, respectively (Fig. 3*A*). This identifies formaldehyde as a direct allosteric effector of FrmR.

**TABLE 1:** **DNA binding affinities and allosteric coupling free energies for FrmR and RcnR** Values were determined from fluorescence anisotropy experiments. The conditions used were as follows: 25 °C, 10 mm HEPES, pH 7.0, 60 mm NaCl, 240 mm KCl with the addition of 5 mm EDTA for titrations with apoprotein or 5 μm NiCl_2_ or CoCl_2_ for metal-loaded titrations. RcnR was incubated with 1.2 molar eq of NiCl_2_ or CoCl_2_ per monomer, as indicated.

Protein	Effector	*K*_DNA_*^a^*
		*m*
FrmR	Apo*^b^*	9.9 ± 0.3 × 10^−8^
FrmR^C35A^	Apo	1.6 ± 0.2 × 10^−7^
FrmR^P2S^	Apo	1.5 ± 0.2 × 10^−7^
RcnR	Apo	1.5 ± 0.8 × 10^−7^
RcnR	Ni(II)	≥5.9 ± 1.3 × 10^−6^
RcnR	Co(II)	≥1.5 ± 0.2 × 10^−5^
RcnR^S2P^	Apo	1.6 ± 0.1 × 10^−7^

*^a^* Data were fit to a model describing two nondissociable tetramers of FrmR or RcnR binding with equal affinity to *frmRA*Pro or *rcnRA*Pro, respectively.

*^b^* Determined previously under the same conditions (50).

The ability of FrmR to respond directly to formaldehyde opens up the possibility that other aldehydes or related alcohols may also act as allosteric effectors. To test this hypothesis, DNA binding was monitored in the presence of acetaldehyde (CH_3_HC=O), which differs from formaldehyde by replacement of a carbonyl-bonded proton with a methyl group. Inclusion of 20 μm acetaldehyde did not weaken the DNA binding affinity of FrmR (Fig. 3*A*), and expression from P*_frmRA_-frmR* was not derepressed when *Salmonella* cells were exposed to MNIC acetaldehyde (Fig. 3*B*). Similarly, FrmR did not respond to ethanol, methanol, 1-butanol, 1-propanol, and 2-propanol *in vivo* (Fig. 3*B*). Together, these data show that the response of FrmR demonstrates specificity for formaldehyde over other organic molecules and suggest that metal ions are not required to transduce the formaldehyde signal to FrmR *in vivo*.

##### Structure of FrmR^E64H^ and Visualization of Its Inferred Metal (Zn(II)/Co(II))-sensing Site

To identify the sensory site(s) of FrmR^E64H^ for metal and formaldehyde, diffraction quality crystals were generated, and an x-ray crystal structure was determined to 2.19 Å resolution (Fig. 4*A*). FrmR^E64H^ exists as a homotetrameric assembly composed of a dimer of dimers as observed for the structurally characterized metal-sensing regulator CsoR (31, 42–44) (Fig. 4*A*). FrmR^E64H^ has a kink (∼45º) in helix α3, not observed in (any) CsoR structure, which is enabled by Gly^83^, a residue specific to *Salmonella* FrmR (distinct from Ile^83^ in *E. coli* FrmR). The electrostatic surface potential highlights a region of positive potential composed of positively charged residues from helices α1 and α2 within a single monomer subunit (Fig. 4*B*). This region (as suggested for metal sensor CsoR (31, 43, 45)) is anticipated to enable binding of FrmR^E64H^ to the *frmRA* operator-promoter, although the precise nature of the protein-DNA interactions for any DUF156 member are unknown. The *Salmonella frmRA* operator-promoter comprises a C_6_ tract flanked by a T/A-rich inverted repeat (Fig. 2*D*). The requirement of these inverted repeats for FrmR and FrmR^E64H^ binding was investigated by fluorescence anisotropy using a modified *frmRA*Pro dsDNA fragment in which one flanking repeat had been mutated (*frmRA*Pro*) (Figs. 2*D* and 4*C*). No binding of FrmR or FrmR^E64H^ to *frmRA*Pro* (10 nm) was detected, indicating a considerably weaker DNA binding affinity (>10^−5^ m) than determined for *frmRA*Pro. This demonstrates that the T/A-rich inverted repeat is required for tight affinity (physiologically relevant) DNA binding to the *frmRA* operator-promoter. The *frmRA* operator-promoter supports binding of two FrmR (or FrmR^E64H^) tetramers (50), and these data are consistent with obligatory tetramer interaction with the *frmRA*Pro inverted repeat.

A candidate metal-binding site of FrmR^E64H^ is formed by the side chains of His^60^ and His^64^ from one subunit and Cys^35^ from the second subunit within the dimeric assembly (Fig. 4*D*). These residues match the *XYZ* motif required for metal binding in related metal sensors RcnR, CsoR, and InrS, and this was the rationale behind the Glu^64^ → His substitution (31, 39, 45, 46, 50). To investigate the role of Cys^35^ and His^60^ in metal binding, site-directed mutants FrmR^C35A^ and FrmR^H60L^ were generated and assayed for their ability to bind Zn(II). Following preincubation with excess ZnCl_2_, neither variant retained Zn(II) during size exclusion chromatography in contrast to wild type FrmR and FrmR^E64H^, which each co-migrate with 1 molar eq of Zn(II) (Fig. 4*E*) (50). This indicates that the affinities of FrmR^C35A^ and FrmR^H60L^ for Zn(II) are considerably weaker than wild type FrmR and implicates these residues in Zn(II) (and by inference Co(II)) coordination. Candidate residues for a fourth ligand required for the tetrahedral coordination geometry observed for Co(II) and inferred for Zn(II) (50) include His^3^ (denoted position *W* in RcnR (46, 61)), Asp^63^, the amino terminus, or solvent (Fig. 4*D*).

##### Proposed Formaldehyde Sensory Site and Reaction Mechanism

**FIGURE 5.**
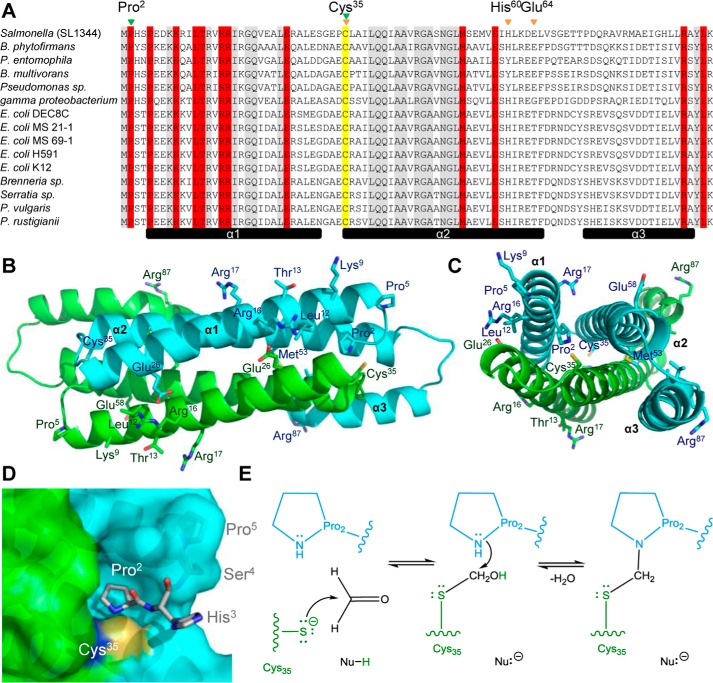
**Conservation of residues in the DUF156 FrmR subgroup and proposed formaldehyde-sensing site.** *A*, alignment of *Salmonella* FrmR with nonredundant UniProtKB DUF156 sequences previously attributed to the FrmR subgroup (45). Organism details and UniProtKB identifiers are outlined under “Experimental Procedures.” Highlighted in *gray* are residues conserved in both FrmR and RcnR subgroups. Highlighted in *red* are residues conserved in the FrmR but not RcnR subgroup. Highlighted in *yellow* is the invariant cysteine present in all DUF156 proteins. The secondary structure elements of the FrmR^E64H^ crystal structure are shown *below* (*black bars*). The inferred Zn(II)/Co(II)-sensing site is identified by *orange arrows*. The proposed formaldehyde sensing site is identified by *green arrows. B* and *C*, dimeric representation of FrmR^E64H^ with the side chains for Cys^35^ and FrmR subgroup-specific residues *labeled*. Each monomer is *colored* differently (using the *same colors* as in Fig. 3*A*) with secondary structure units *labeled* on the *cyan* subunit. *D*, solvent-accessible surface representation of the proposed formaldehyde-binding site, which comprises Pro^2^ (subunit 1, *cyan*) and Cys^35^ (subunit 2, *green*). *E*, proposed reaction of formaldehyde with FrmR Cys^35^ (*green*) followed by Pro^2^ (*cyan*) (both deprotonated ultimately to water) via an *S*-hydroxymethyl intermediate. The reciprocal reaction with Pro^2^ followed by Cys^35^ via an *N*-methylol intermediate is also possible. In both cases, a methylene bridge (*black*) between the two residues is the final product. The nucleophile(s) responsible for deprotonation of Cys^35^ and Pro^2^ remain unknown.

To define the functional formaldehyde sensory site, residues specifically conserved within the FrmR subgroup of the DUF156 family of transcriptional regulators were identified. Protein sequences previously ascribed to the FrmR subgroup (45) were used to generate a multiple-sequence alignment with *Salmonella* FrmR (Fig. 5*A*). Twelve residues are conserved within the FrmR subgroup but absent from the closely related Ni(II)/Co(II)-sensing RcnR subgroup. Two-thirds of the conserved residues are clustered in helix α1 based on the structure of FrmR^E64H^ (Fig. 5, *A–C*). Sensing of formaldehyde may proceed via reaction with Cys^35^, also implicated in the FrmR metal site (Fig. 4, *D* and *E*) due to its conservation in all characterized DUF156 members. Formation of an *S*-formyl adduct at this Cys-thiol followed by reaction with a primary amine has been suggested as a possible reaction mechanism of FrmR with formaldehyde (30). The pyrrolidine side chain of proline residue 2 (α1) is in close proximity (3.0–3.2 Å in the four independent locations within the tetrameric structure) to Cys^35^ from α2′ (Fig. 5, *B* and *C*, and supplemental Fig. S1*A*). A second FrmR-specific proline (Pro^5^) acts to terminate helix α1 and positions the amino terminus of FrmR^E64H^ adjacent to Cys^35^ (Fig. 5*B*). Pro^2^ is the first residue identified in the FrmR^E64H^ structure and is positioned in a pocket at the dimer interface, leaving no space (and no observed electron density) for the amino-terminal methionine predicted by the primary sequence (Fig. 5*D* and supplemental Fig. S1*A*). The amino-terminal region has been implicated in the coordination of Ni(II)/Co(II) by RcnR and of Ni(II) by InrS (61, 62). In the absence of Met^1^, the terminal secondary amine of Pro^2^ and a Cys^35^-thiolate are both ideal candidates for nucleophilic addition to formaldehyde (Fig. 5, *D* and *E*) (63, 64). Either reaction with Pro^2^ followed by Cys^35^ via an *N*-methylol intermediate or reciprocally via an *S*-hydroxymethyl intermediate is plausible (Fig. 5*E*). In both cases, the end product would be a methylene bridge between the two residues, requiring a 1:1 formaldehyde/FrmR monomer (4 possible sites/tetramer) reaction stoichiometry.

**FIGURE 6.**
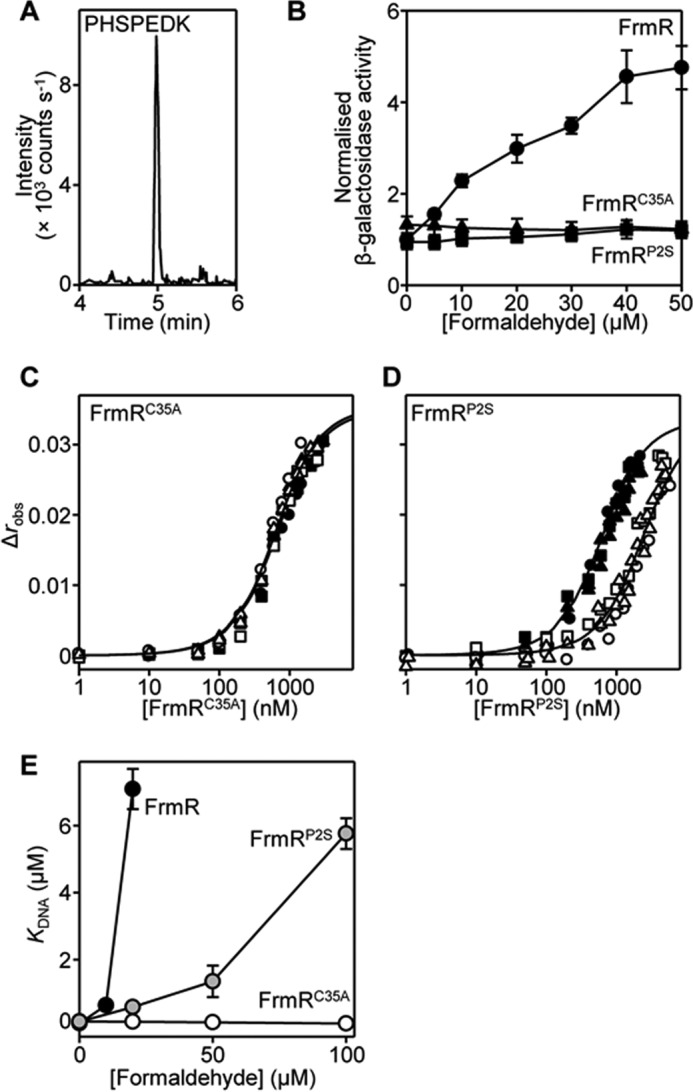
**Pro^2^ and Cys^35^ are required for formaldehyde sensing by FrmR.** *A*, LC-MS chromatogram following multiple-reaction monitoring of purified FrmR. Ion transition 405.19/488.24 is for analyte PHSPEDK. *B*, β-galactosidase activity in SL1344 containing P*_frmRA_-frmR* (*circles*), P*_frmRA_-frmR^C35A^* (*triangles*), or P*_frmRA_-frmR^P2S^* (*squares*) fused to *lacZ* grown to mid-exponential phase in M9 minimal medium in the presence of formaldehyde (MNIC = 50 μm). Values are means of three biological replicates (each performed in triplicate) with S.D. *C* and *D*, anisotropy change upon titration of a limiting concentration of *frmRA*Pro (10 nm) with FrmR^C35A^ (*C*) or FrmR^P2S^ (*D*) in the presence of 5 mm EDTA (*closed symbols*) and with the addition of 20 μm formaldehyde (*open symbols*). Data were fit to a model describing a 2:1 protein tetramer (nondissociable)/DNA stoichiometry (binding with equal affinity) (50, 86), and *lines* represent simulated curves produced from the average (apparent) *K*_DNA_ determined across the experimental replicates shown. *Symbol shapes* represent individual experiments. *E*, apparent *K*_DNA_ values of FrmR (*black symbols*), FrmR^P2S^ (*gray symbols*), and FrmR^C35A^ (*open symbols*) with increasing formaldehyde concentration. Values are means of three replicates with S.D. (*error bars*).

The cleavage of FrmR Met^1^ was examined by multiple-reaction monitoring MS using purified FrmR. Amino-terminal peptide PHSPEDK was detected, confirming that FrmR is a substrate for methionine aminopeptidase (Fig. 6*A*). To investigate the requirement of Cys^35^ and Pro^2^ for formaldehyde sensing, transcriptional fusions of P*_frmRA_-frmR^C35A^* and P*_frmRA_-frmR^P2S^* with *lacZ* were generated, introduced into *Salmonella*, and compared with wild type (P*_frmRA_-frmR*). Expression from P*_frmRA_-frmR* is derepressed by exposure of cells to formaldehyde in a concentration-dependent manner (Fig. 6*B*). Repression of P*_frmRA_* is retained following mutation of either Cys^35^ or Pro^2^, but derepression in response to formaldehyde is completely abolished (Fig. 6*B*). To confirm that formaldehyde was unable to act as an allosteric effector of these FrmR variants, the DNA binding properties of FrmR^C35A^ and FrmR^P2S^ were characterized using fluorescence anisotropy (Fig. 6, *C* and *D*). Titration of *frmRA*Pro with apo-FrmR^C35A^ or apo-FrmR^P2S^ in the presence of excess EDTA revealed *K*_DNA_ values comparable with wild type FrmR and consistent with the observed repression by both mutants *in vivo* (*K*_DNA_^apo-FrmRC35A^ = 1.6 ± 0.2 × 10^−7^ m and *K*_DNA_^apo-FrmRP2S^ = 1.5 ± 0.2 × 10^−7^ m) (Fig. 6, *C* and *D*, and Table 1). However, in contrast to wild type FrmR (Fig. 3*A*), DNA binding by FrmR^C35A^ was unaffected by the presence of 20 μm formaldehyde, indicating a loss of formaldehyde reactivity (Fig. 6*C*). The reactivity of FrmR^P2S^ to formaldehyde was significantly decreased compared with wild type FrmR with apparent DNA binding affinity weaker than apo-FrmR^P2S^ by only ∼4-fold in the presence of 20 μm formaldehyde (compared with ≥70-fold for wild type FrmR (Fig. 3*A*)) (Fig. 6*D*). Consequently, the FrmR^C35A^ substitution impairs formaldehyde reactivity more severely than FrmR^P2S^ (Fig. 6*E*). The proposed mechanism (1:1 formaldehyde/FrmR stoichiometry (Fig. 5*E*)) and observed DNA binding by FrmR implies an affinity at the allosteric site(s) of FrmR for formaldehyde in the 10–20 μm range, whereas formaldehyde affinities of FrmR^C35A^ and FrmR^P2S^ variants are inferred to be ≫100 μm and >50 μm, respectively (Fig. 6*E*). These data implicate Cys^35^ and Pro^2^ in formaldehyde-mediated derepression *in vivo* and impaired DNA binding *in vitro* (Fig. 6). The Zn(II)/Co(II) site also requires Cys^35^ (Fig. 4, *D* and *E*), implying overlap between the two effector sensory sites.

##### FrmR Is More Sensitive to Formaldehyde than RcnR

**FIGURE 7.**
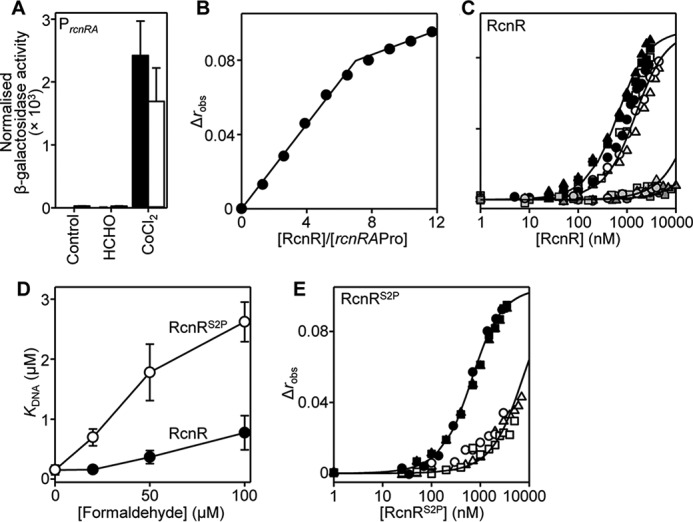
**RcnR is less formaldehyde-responsive but RcnR^S2P^ gains reactivity.** *A*, β-galactosidase activity in SL1344 containing *rcnR-*P*_rcnRA_* (*solid bars*) or *rcnR^S2P^-*P*_rcnRA_* (*open bars*) fused to *lacZ* grown to mid-exponential phase in M9 minimal medium in the absence or presence of MNIC formaldehyde (50 μm) or CoCl_2_ (1 μm). Values are means of three biological replicates (each performed in triplicate) with S.D. (*error bars*). *B* and *C*, anisotropy change upon titration of a high concentration of *rcnRA*Pro (2.5 μm) with RcnR in the presence of 5 mm EDTA (*B*) or a limiting concentration of *rcnRA*Pro (10 nm) with RcnR in the presence of 5 mm EDTA (*black symbols*) and with the addition of 50 μm formaldehyde (*open symbols*) or Ni(II)-RcnR (*light gray symbols*) and Co(II)-RcnR (*dark gray symbols*) in the presence of 5 μm NiCl_2_ or 5 μm CoCl_2_, respectively (*C*). *Symbol shapes* represent individual experiments. Data were fit to a model describing a 2:1 protein tetramer (nondissociable)/DNA stoichiometry (binding with equal affinity) (50, 86), and *lines* represent simulated curves produced from the average (apparent) *K*_DNA_ determined across the experimental replicates shown. *D*, apparent *K*_DNA_ values of RcnR (*black symbols*) and RcnR^S2P^ (*open symbols*) with increasing formaldehyde concentration. Values are means of three replicates with S.D. *E*, as described in *C* except with RcnR^S2P^ in the presence of 5 mm EDTA (*black symbols*) and with the addition of 50 μm formaldehyde (*open symbols*).

Although FrmR shares sequence similarities with Ni(II)/Co(II)-sensing RcnR (30, 32, 45, 46), expression from *Salmonella rcnR*-P*_rcnRA_* fused to *lacZ* is not derepressed by formaldehyde *in vivo* (Fig. 7*A*). Analysis of the *rcnR-rcnA* intergenic region identified two putative RcnR DNA-binding sequences in the target RcnR operator-promoter (supplemental Table S1). The interaction of RcnR with a fluorescently labeled double-stranded DNA fragment containing these sequences, *rcnRA*Pro, was monitored by fluorescence anisotropy. The stoichiometry of *Salmonella* RcnR binding to *rcnRA*Pro was first confirmed by titration of RcnR into a relatively high concentration of DNA (2.5 μm) with saturation observed at 8 molar eq of RcnR (monomer) consistent with binding of two tetramers (one per site) (Fig. 7*B*) as observed for *E. coli* RcnR (65). A limiting concentration of DNA (10 nm) and a model describing the binding of two non-dissociable RcnR tetramers per DNA molecule were subsequently used to determine the *K*_DNA_ of RcnR and *rcnRA*Pro as 1.5 ± 0.8 × 10^−7^ m for apo-RcnR (Fig. 7*C* and Table 1). As predicted, titration of *rcnRA*Pro with either Ni(II)-RcnR or Co(II)-RcnR dramatically weakened DNA binding (Fig. 7*C* and Table 1); *K*_DNA_^Ni(II)-RcnR^ ≥ 5.9 ± 1.3 × 10^−6^ m and *K*_DNA_^Co(II)-RcnR^ ≥ 1.5 ± 0.2 × 10^−5^ m. The allosteric coupling free energy (Δ*G*_C_), which couples effector binding to DNA binding (66–68), is calculated to be ≥+2.2 ± 0.2 and ≥+2.7 ± 0.2 kcal mol^−1^ for Ni(II)- and Co(II)-RcnR, respectively. Conversely, DNA binding by RcnR is unaffected by the inclusion of 20 μm formaldehyde (Fig. 7*D*), a concentration that weakens FrmR DNA binding by ≥70-fold (Fig. 6*E*). Importantly, these data establish a degree of specificity of FrmR over RcnR for formaldehyde.

##### Generation of an RcnR^S2P^ Variant with Enhanced Response to Formaldehyde in Vitro

Increasing the concentration of formaldehyde during fluorescence anisotropy to 50 and 100 μm does impair binding of RcnR to *rcnRA*Pro by ∼2.5- and ∼5-fold (relative to apo-RcnR), respectively (Fig. 7, *C* and *D*), providing an assay to monitor changes in RcnR formaldehyde reactivity. Introduction of the proposed formaldehyde sensing site of FrmR into *Salmonella* RcnR was achieved by mutation of Ser^2^ to Pro^2^ (Cys^35^ is conserved in both proteins). Titration of RcnR^S2P^ into *rcnRA*Pro (10 nm) confirms that this variant binds *rcnRA*Pro with equal affinity to wild type RcnR (*K*_DNA_^apo-RcnRS2P^ =1.6 ± 0.1 × 10^−7^ m) (Fig. 7*E* and Table 1). DNA binding by RcnR^S2P^ was subsequently assessed in the presence of formaldehyde; inclusion of 50 and 100 μm formaldehyde weakened the apparent DNA affinity of RcnR^S2P^ by ∼11- and ∼17-fold, respectively (Fig. 7, *D* and *E*). Thus, the single Ser^2^ → Pro point mutation generates an RcnR variant with increased reactivity to formaldehyde compared with wild type RcnR (Fig. 7*D*). However, this increase was not sufficient to gain formaldehyde sensing by *rcnR*^S2P^-P*_rcnRA_ in vivo* (Fig. 7*A*). Repression from P*_rcnRA_* under control conditions (without inducer), combined with an observed cobalt responsiveness, confirmed that the expressed RcnR^S2P^ was functional (Fig. 7*A*). It is inferred that the threshold for formaldehyde detection is not met by RcnR^S2P^ *in vivo*.

##### Glutathione Inhibits Formaldehyde Sensing

**FIGURE 8.**
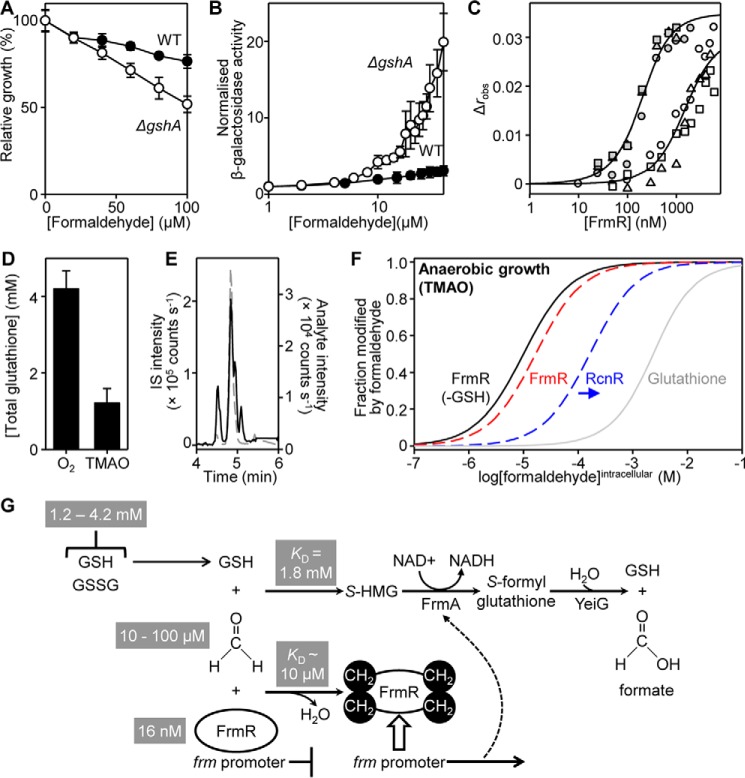
**The relationship between glutathione and formaldehyde sensing by FrmR.** *A*, survival of wild type *Salmonella* SL1344 (*solid circles*) or Δ*gshA* (*open circles*) grown to mid-exponential phase in M9 minimal medium in the presence of formaldehyde. Values are means of three biological replicates (each performed in triplicate) with S.D. (*error bars*). *B*, β-galactosidase activity of SL1344 (*solid symbols*) or Δ*gshA* (*open symbols*) containing P*_frmRA_-frmR* fused to *lacZ* grown to mid-exponential phase in M9 minimal medium in the presence of formaldehyde (MNIC = 50 and 20 μm for wild type and Δ*gshA*, respectively; see supplemental Fig. S2 for corresponding growth data). Values are means of at least three biological replicates (each performed in triplicate) with S.D. *C*, anisotropy change upon titration of a limiting concentration of *frmRA*Pro (10 nm) with FrmR in the presence of 5 mm EDTA and 800 μm GSH in the absence (*gray symbols*) or presence (*open symbols*) of 20 μm formaldehyde. *Symbol shapes* represent individual experiments. Data were fit to a model describing a 2:1 protein tetramer (nondissociable)/DNA stoichiometry (binding with equal affinity) (50, 86), and *lines* represent simulated curves produced from the average (apparent) *K*_DNA_ determined across the experimental replicates shown. *D*, intracellular glutathione concentration in *Salmonella* cells following growth to exponential phase in M9 minimal medium aerobically (O_2_) or anaerobically with TMAO as an alternative electron acceptor. Values are means of three biological replicates with S.D. *E*, representative (*n* = 3) LC-MS chromatograms of ion transitions detected in mid-logarithmic *Salmonella* SL1344 cells under aerobic growth conditions. Transitions are for analyte GQVEALER (*solid black line*) or labeled internal standard (*IS*) (GQVEALER[^13^C_6_,^15^N_4_], where R[^13^C_6_,^15^N_4_] represents ^13^C,^15^N-labeled arginine) (*dashed gray line*). *F*, fractional modification by formaldehyde of FrmR (*solid black line*), GSH (*solid gray line*), or FrmR (*dashed red line*) and RcnR (*dashed blue line*; tighter limit as indicated by the *blue* arrow) in the presence of GSH in *Salmonella* cells grown anaerobically with TMAO. Formaldehyde affinities of 10^−5^, 10^−4^ (tighter limit), and 1.77 × 10^−3^ m (73) were used for FrmR, RcnR, and GSH, respectively. Intracellular abundance was determined for FrmR (16.1 ± 0.2 nm) and GSH (1.2 ± 0.4 mm) and estimated for RcnR, as described under “Experimental Procedures.” *G*, the role of glutathione in formaldehyde detoxification and sensing in *Salmonella*. In the absence of effector, *Salmonella* FrmR represses the *frm* promoter. Formaldehyde directly modifies FrmR (*reaction 1* in Fig. 1) via a deduced intersubunit methylene bridge between Pro^2^ and Cys^35^ (Fig. 5, up to four per tetramer) derepressing *frm* expression. GSH inhibits formaldehyde detection (*reaction 3* in Fig. 1), and despite the high [glutathione], the affinity of FrmR for formaldehyde is sufficiently tight relative to GSH to enable expression of FrmA to coincide with the appearance of its substrate. The *Salmonella frm* operon lacks *frmB*, and YeiG may catalyze the final detoxification step. *S-HMG*, *S*-(hydroxymethyl)glutathione.

The substrates of the FrmR-regulated alcohol dehydrogenase from *Salmonella* (FrmA) are predicted to be the formaldehyde and nitrosylated adducts of GSH, *S*-(hydroxymethyl)glutathione and *S*-nitrosoglutathione, respectively, by analogy to *E. coli* (Fig. 2*B*) (47, 69). Despite the evidence that DNA binding by FrmR is directly weakened by formaldehyde *in vitro* (Fig. 3*A*), glutathione adducts of formaldehyde might represent the predominant available species during formaldehyde stress conditions. Notably, glutathione has been shown to act positively on metal detection by FrmR^E64H^ *in vivo*, suggesting that the protein may interact with glutathione adducts (Fig. 1) (50). Deletion of *gshA*, encoding γ-glutamate-cysteine ligase (70), required for the first step in glutathione biosynthesis, renders *Salmonella* more sensitive to exogenous formaldehyde compared with the wild type strain (Fig. 8*A*), as expected if (as in *E. coli*) glutathione is required for formaldehyde detoxification in *Salmonella* by formation of *S*-(hydroxymethyl)glutathione. However, formaldehyde-mediated derepression of P*_frmRA_-frmR* was not impaired in Δ*gshA* cells (Fig. 8*B*), indicating that formation of formaldehyde-glutathione adducts is not an absolute requirement for FrmR responsiveness to formaldehyde *in vivo*. Indeed, expression levels from P*_frmRA_-frmR* were higher in Δ*gshA* than in wild type, at equivalent exogenous formaldehyde concentrations (Fig. 8*B*), consistent with FrmR detecting increased formaldehyde accumulation in the cytosol of Δ*gshA* cells, due to reduced FrmA activity and/or due to glutathione acting negatively on the modification of FrmR by formaldehyde.

Binding of FrmR to *frmRA*Pro was monitored by fluorescence anisotropy in the presence of 800 μm GSH. GSH alone has a minimal but detectable effect on DNA binding by apo-FrmR (∼2.5-fold tighter) (Fig. 8*C*). The ability of FrmR to respond to formaldehyde in the presence of GSH was assessed by subsequent titration of FrmR into *frmRA*Pro in the presence of both formaldehyde (20 μm) and excess GSH (800 μm). The apparent DNA affinity of FrmR was weaker (relative to FrmR and GSH alone, without formaldehyde) by ∼9-fold, but critically, the magnitude of the response by FrmR to formaldehyde is diminished by GSH (compare *open symbols* in Figs. 3*A* and 8*C*). These data show that GSH competes with FrmR for formaldehyde rather than contributing toward its reactivity. How then can FrmR detect free formaldehyde *in vivo*, since glutathione is expected to be in a large molar excess? Under aerobic conditions, the intracellular glutathione concentration in *Salmonella* cells was determined to be 4.2 ± 0.5 mm (Fig. 8*D*), whereas the abundance of FrmR was 9.7 ± 2.6 tetramers/cell (16.1 ± 0.2 nm), as determined by quantitative mass spectrometry (Fig. 8*E* and supplemental Table S2). Repression by *E. coli* FrmR is alleviated during TMAO-mediated anaerobic respiration, probably due to TMAO demethylase activity and intracellular formaldehyde generation (15). The concentration of glutathione in *Salmonella* drops to 1.2 ± 0.4 mm when cells are grown anaerobically using TMAO as an alternative electron acceptor (Fig. 8*D*). These data have been used to model formation of *S*-(hydroxymethyl)glutathione and the modification of FrmR as a function of [formaldehyde], with implications for the species detected by FrmR *in vivo* discussed below (Fig. 8*F*).

## Discussion

Detection of metals and formaldehyde by *Salmonella* FrmR^E64H^ is retained when the sensor is expressed in a heterologous *E. coli* host (Fig. 2). Zn(II) is not required to transduce the formaldehyde signal *in vivo* because formaldehyde directly allosterically activates wild type *Salmonella* FrmR *in vitro* (Fig. 3). The allosteric response to organic molecules is specific to formaldehyde and not acetaldehyde *in vitro* and *in vivo* (Fig. 3). Deduced sensory sites for Zn(II)/Co(II) and for formaldehyde overlap with both effectors requiring Cys^35^ (Figs. 4 and 5). Substitution of either Cys^35^ or Pro^2^ decreases the reactivity of FrmR to formaldehyde *in vitro* and abolishes sensing *in vivo* (Fig. 6). The sensory site of FrmR is more reactive to formaldehyde than the related Ni(II)/Co(II) sensor RcnR *in vitro*, and RcnR does not respond to formaldehyde *in vivo* (Fig. 7). Introduction of the deduced formaldehyde sensory site to generate RcnR^S2P^ confers increased reactivity to formaldehyde *in vitro* (Fig. 7). Although *S*-(hydroxymethyl)glutathione is a substrate for FrmA, free formaldehyde is the allosteric effector of FrmR, and glutathione competes with FrmR for formaldehyde both *in vitro* and *in vivo* (Fig. 8).

The unexpected ability of Zn(II) and Cu(I) to weaken *Salmonella* FrmR *K*_DNA_ *in vitro* (50) raised the possibility that metals might act as signal transducers of intracellular formaldehyde accumulation (Fig. 1). Moreover, there is precedence for a Zn(II)-dependent alcohol dehydrogenase being regulated in response to Zn(II) by Zap1 (zinc-responsive activator protein) transcription factor as a Zn(II)-sparing mechanism in yeast (71, 72). FrmR-regulated glutathione-dependent formaldehyde dehydrogenase, FrmA, similarly requires Zn(II) for activity (47). However, here we eliminate the requirement of Zn(II) during FrmR-mediated derepression of *frmRA* because formaldehyde is shown by fluorescence anisotropy to be a direct allosteric effector of FrmR (Figs. 2 (*E* and *F*) and 3). The related metal sensor RcnR (which shares 40% identity with FrmR) is less reactive to formaldehyde by at least an order of magnitude (Figs. 3*A*, 6*E*, and 7 (*C* and *D*)). Candidate effector sensory sites for formaldehyde and Zn(II)/Co(II) were identified by structural characterization of FrmR^E64H^ (Figs. 4 and 5) and shown by site-directed mutagenesis to each require Cys^35^ (Figs. 4*E* and 6 (*B*, *C*, and *E*)). We show that an FrmR-specific amino terminus, Pro^2^, is also required to react with formaldehyde and propose formation of an interdimer methylene bridge between the two residues (Figs. 5 and 6 (*D* and *E*) and supplemental Fig. S1*A*). Introduction of such a cross-link would only alter the distance between Cys^35^ and Pro^2^ by ∼+0.5 Å relative to the crystal structure. Future studies should aim to visualize the proposed methylene bridge and the nature of allosteric coupling between formaldehyde modification and DNA binding. Indeed, such coupling is yet to be characterized for any DUF156 family member. The unique (to date) Gly^83^ in *Salmonella* FrmR and the resulting kink in α3 may make this protein distinct.

Glutathione is not required for FrmR to respond to formaldehyde *in vivo* (Fig. 8*B*). Rather than aid detection, glutathione competes with FrmR for formaldehyde *in vitro* and inhibits the response *in vivo* (Fig. 8, *B* and *C*). In contrast, although glutathione acts positively toward cobalt detection by FrmR^E64H^ *in vivo* (50), the present data argue against a suggestion that FrmR^E64H^ preferentially detects cobalt due to its interaction with glutathione conjugates (Fig. 8). Because glutathione is such an abundant biomolecule (Fig. 8*D*), *S*-(hydroxymethyl)glutathione (the substrate for FrmA) might be expected to predominate over formaldehyde in a cell. However, the affinity of FrmR for formaldehyde is inferred to be ∼10^−5^ m from measured DNA affinities (Fig. 6*E*), substantially tighter than the affinity for formation of *S*-(hydroxymethyl)glutathione from formaldehyde and GSH (1.77 × 10^−3^ m) (73) (Fig. 8*G*). Thus, although GSH is at least 5 orders of magnitude more abundant than FrmR (determined to be 16.1 ± 0.2 nm; Fig. 8*E* and supplemental Table S2), FrmR will nonetheless be >85% modified by formaldehyde at cellular [formaldehyde], where only 4% of the GSH pool is in the *S*-(hydroxymethyl)glutathione form (Fig. 8 (*F* and *G*), *dashed red line* and *gray line*). Crucially, this means that expression of *frmA* will be derepressed as cellular *S*-(hydroxymethyl)glutathione begins to accumulate (Fig. 8*F*). Importantly, the ability of FrmR to respond to formaldehyde directly prevents [formaldehyde] from rising to levels where cross-linking of other cellular proteins (*e.g.* RcnR) (Fig. 8*F*, *blue dashed line*) or significant depletion of the GSH pool would occur.

The *Salmonella* and *E. coli frm* operons are distinct (Fig. 2), which could reflect requirements specific to pathogenicity with a suggestion that formaldehyde generation may arise following the macrophage respiratory burst (2). Consistent with this, the *frmRA* locus is known to be up-regulated during intracellular survival of *Salmonella* within macrophages (74, 75). Unlike *E. coli*, the *Salmonella frm* locus does not present a complete formaldehyde detoxification pathway (or recycling of GSH) due to the lack of *frmB*. YeiG, capable of catalyzing the formation of formate and GSH from *S*-formylglutathione in *E. coli* (27), is also present in *Salmonella* (Fig. 2*B*) and is an obvious candidate to function in the absence of FrmB (Fig. 8*G*). Furthermore, *yeiG*, which is not FrmR-regulated, is co-expressed with genes encoded by the *Salmonella* pathogenicity island-2 (SPI-2), notable for being up-regulated and absolutely required during replication within eukaryotic cells (74–76). It is formally possible that differences may emerge between the effectors and sensory sites of *Salmonella versus E. coli* FrmR (*e.g.* residues surrounding sensory sites, specificities to organic molecules, metals as allosteric effectors, nature of allostery) that reflect the demands for survival in the distinctive niches inhabited by each organism.

The FrmR sensory site is more reactive to formaldehyde than, for example, RcnR. The amino terminus of mature FrmR becomes a pyrrolidine secondary amine from Pro^2^, in contrast to the primary amino group of RcnR (from Ser^2^). Consequently, the nucleophilic reactivity of the FrmR amino terminus is predicted to be greater than RcnR (63, 64) and more able to undergo nucleophilic addition to the formaldehyde carbonyl group (Fig. 5). A Cys^35^-thiolate in both proteins also presents a particularly nucleophilic group capable of this reaction (Fig. 5) (63, 64). We propose that the presence of this reactive pair in FrmR would allow formation of an interdimer cross-link (Fig. 5). Consistent with this, Pro^2^ and Cys^35^ are required for formaldehyde detection by FrmR (Fig. 6), and creation of the proposed formaldehyde site in RcnR^S2P^ increases sensitivity to formaldehyde *in vitro* (Fig. 7). However, mutation of the RcnR amino terminus alone is not sufficient to confer the same degree of reactivity exhibited by FrmR; therefore, it is likely that additional residues optimize formation of a cross-link. Most notable is Pro^5^, another FrmR-specific residue that terminates helix α1 and may confer a degree of rigidity to the amino terminus, positioning Pro^2^ into the sensory site adjacent to Cys^35^.

Effector selectivity of DUF156 family transcriptional repressors can be changed by relatively modest sequence alterations. Conservation of a cysteine at the effector site is now confirmed to be common to a formaldehyde-sensing family member (Figs. 5 and 6) as well as the metal sensors. Changing single residues proximal to this active cysteine has 1) increased the ability of RcnR to sense formaldehyde *in vitro* in RcnR^S2P^ (Fig. 7, *D* and *E*), 2) enabled metal sensing *in vivo* by FrmR^E64H^ (Fig. 2*F*) (50), and 3) switched the metal specificity of RcnR^H3E^ *in vivo* (61). Notably, although RcnR^S2P^ is more reactive to formaldehyde than RcnR *in vitro*, it still cannot respond *in vivo* (Fig. 7). Furthermore, FrmR can respond to metal *in vitro* but not *in vivo* (Fig. 2*E*) (50), the latter being achieved by the FrmR^E64H^ variant. In the case of FrmR^E64H^, the threshold for Zn(II) responsiveness *in vivo* was met by a tighter Zn(II) affinity and weaker apo-DNA affinity (relative to wild type FrmR), rendering FrmR^E64H^ competitive relative to cognate Zn(II) sensors, ZntR and Zur (50). FrmR responds to formaldehyde in a cell, placing it above some threshold of reactivity for this effector (Figs. 2*E*, 3*B*, and 8*F*). FrmR^C35A^ and FrmR^P2S^ variants, along with RcnR and RcnR^S2P^, must be below the threshold for formaldehyde sensing (Figs. 6 (*B–E*) and 7 (*C–E*)). Presumably, cells do not survive at [effector] sufficient to trigger such sensors. Among the FrmR-RcnR DUF156 proteins (and yet to be tested for CstR and CsoR), subtle quantitative changes to effector responses tune these sensors above or below different cellular thresholds, and this is sufficient to confer the necessary level of specificity *in vivo*.

## Experimental Procedures

### 

#### 

##### Bacterial Strains and DNA Manipulations

*S. enterica* sv. *Typhimurium* strain SL1344 was used as wild type, and strain LB5010a was used as a restriction-deficient modification-proficient host for DNA manipulations (50). Deletion derivatives of SL1344 lacking *frmR* and *gshA* were generated previously (50). *E. coli* strains BW25113Δ*frmR*, in which the *frmR* coding sequence is disrupted by a *kan*^R^ cassette (77), was used for β-galactosidase assays. This was a gift from D. Weinkove (Durham University). *E. coli* strain DH5α was used for routine cloning, and strain BL21(DE3) was used for recombinant protein overexpression. Bacteria were cultured aerobically (with shaking) at 37 °C in LB medium or M9 minimal medium (78), supplemented with thiamine (0.001%, w/v) and either l-histidine (20 μg ml^−1^) for *Salmonella* or 1 μm C_6_H_5_FeO_7_ for *E. coli*. Carbenicillin (100 μg ml^−1^), kanamycin (25 μg ml^−1^), and TMAO (40 mm) were added where appropriate. Cells were transformed to antibiotic resistance as described (78, 79). For glutathione quantification under aerobic and anaerobic conditions, glucose was replaced with glycerol as a non-fermentable carbon source. For survival assays, overnight cultures were grown in M9 minimal medium, diluted 1:50 into fresh medium in 14-ml culture tubes containing the indicated concentrations of formaldehyde, and grown to mid-logarithmic phase. Growth was assessed by measuring absorbance at 600 nm and calculating the percentage survival compared with the control condition for each strain. Experiments were performed in triplicate on at least three separate occasions. Generated plasmid constructs were checked by sequence analysis. Primers are listed in supplemental Table S1.

##### Bioinformatic Analysis

Fourteen FrmR and nine RcnR non-redundant primary amino acid sequences identified in (45) and still present in UniProtKB (80) were aligned with the *S. enterica* serovar Typhimurium SL1344 FrmR sequence (UniProtKB identifier: A0A0H3NLH8) using the PRALINE multiple-sequence alignment tool (81). UniProtKB identifiers for the FrmR sequences were as follows: B2SZZ0, *Burkholderia phytofirmans* (strain DSM 17436/PsJN); Q1IAA5, *Pseudomonas entomophila* (strain L48); B9BFA7, *Burkholderia multivorans* CGD1; F0DZ53, *Pseudomonas* sp. (strain TJI-51); B5JUQ3, Gammaproteobacterium HTCC5015; H4ZQC4, *E. coli* DEC8C; D8A2B2, *E. coli* (strain MS 21–1); D7ZJL9, *E. coli* MS 69–1; F4VAD6, *E. coli* H591; P0AAP3 (*blue*), *E. coli* (K12); G7LSK1, *Brenneria* sp. EniD312; I0QLA2, *Serratia* sp. M24T3; Q8KKB0, *Proteus vulgaris*; D1P3L2, *Providencia rustigianii* DSM 4541. Residues present in FrmR but not RcnR sequences were identified as FrmR-specific. For three sequences (D8A2B2, D7ZJL9, and F4VAD6) amino-terminal residues annotated by UniprotKB were not predicted to be coding residues using the NCBI (National Center for Biotechnology Information) database and were removed. Phylogenetic analysis was performed using ClustalW2 phylogeny (82); *E. coli* FrmR sequences except for *E. coli* K12 were removed. Distance values relate to the number of substitutions as a proportion of the length of the alignment (excluding gaps). Amino acid sequence identities were determined using Clustal Omega (83).

##### Generation of Promoter-lacZ Fusion Constructs and β-Galactosidase Assays

Promoter-*lacZ* fusions P*_frmRA_-frmR*, P*_frmRA_-frmR^E64H^*, and *rcnR*-P*_rcnRA_* have been described previously (50). Subcloning vector pGEM-T containing either the P*_frmRA_-frmR* or *rcnR*-P*_rcnRA_* DNA fragment (50) was used as a template for site-directed mutagenesis via the QuikChange® protocol (Stratagene) using primers 1 and 2 to generate P*_frmRA_-frmR^P2S^*, primers 3 and 4 to generate P*_frmRA_-frmR^C35A^*, or primers 5 and 6 to generate *rcnR^S2P^*-P*_rcnRA_* (primers listed in supplemental Table S1). Digested fragments were cloned into the SmaI/BamHI site of pRS415 (84). Constructs were introduced into *E. coli* strain BW25113Δ*frmR* as appropriate or *Salmonella* strain LB5010a before SL1344 (and derivatives). β-Galactosidase assays were performed as described (50, 85), in triplicate, and on at least three separate occasions. Briefly, overnight cultures were grown in M9 minimal medium; diluted 1:50 in fresh medium supplemented with up to MNIC (defined as the maximum concentration that inhibited growth by ∼10%) of metal, alcohol, or aldehyde; and grown to mid-logarithmic phase before assays. MNICs under these growth conditions were 5 μm CoCl_2_, 50 μm ZnCl_2_, 300 mm ethanol, 600 mm methanol, 5 mm 1-butanol, 50 mm 1-propanol, 200 mm 2-propanol, 50 μm formaldehyde, and 3 mm acetaldehyde, with the exception that 1 μm CoCl_2_ was the MNIC for cells expressing *rcnR*-P*_rcnRA_* or *rcnR^S2P^*-P*_rcnRA_*. Time course experiments were performed as described (50) by exposing logarithmic cells to MNIC metal or formaldehyde for 2 h at 25 °C. Where stated, β-galactosidase activity (nmol of *o*-nitrophenol min^−1^ mg of protein^−1^) was normalized to the control data for cells expressing the wild type protein conducted in parallel.

##### Protein Expression and Purification

Vectors for overexpression of FrmR, FrmR^E64H^, and RcnR have been described previously (50). Site-directed mutagenesis was conducted as described above using template pETfrmR and primers 7–12 to generate pETfrmR^P2S^, pETfrmR^C35A^, and pETfrmR^H60L^ or using template pETrcnR and primers 13 and 14 to generate pETrcnR^S2P^. Proteins were expressed and purified as described previously (50). Mutant variants were purified exactly as described for the respective wild type protein. Protein purity was assessed by SDS-PAGE. Anerobic protein stocks (maintained in an anaerobic chamber) were prepared as described and confirmed to be ≥90% reduced and ≥95% metal-free (50). FrmR and variants were stored in 100 mm NaCl, 400 mm KCl, 10 mm HEPES, pH 7.0. RcnR and variants were stored in 200 mm NaCl, 800 mm KCl, 10 mm HEPES, pH 7.0. All *in vitro* experiments were carried out under anaerobic conditions using Chelex-treated and N_2_-purged buffers as described previously (50). Due to the absence of any thiol groups, experiments with FrmR^C35A^ were carried out under aerobic conditions.

##### Inductively Coupled Plasma MS

Anaerobic protein stocks (10–20 μm) or size exclusion chromatography fractions were diluted 10-fold in 2.5% Suprapur HNO_3_ (Merck Millipore). Quantitative analysis of metal content was determined using an XSERIES-2 inductively coupled plasma mass spectrometer (Thermo Fisher Scientific) following calibration with elemental standards that were matrix-matched to the sample by inclusion of an appropriate buffer system.

##### Fluorescence Anisotropy

Fluorescently labeled double-stranded DNA probe, *frmRA*Pro, containing the identified FrmR-binding site has been described previously (50). Complementary single-stranded oligonucleotides 15 (hexachlorofluorescein-labeled) and 16 (containing two identified RcnR-binding sites (32, 65) and flanking oligonucleotides) or 17 (hexachlorofluorescein-labeled) and 18 (*frmRA*Pro but with mutation of one T/A-rich inverted repeat) (supplemental Table S1) were annealed by heating a 10 or 200 μm concentration of each strand in 150 mm NaCl, 10 mm HEPES, pH 7.0, to 95 °C and cooled slowly overnight, to generate *rcnRA*Pro (35 bp) or *frmRA*Pro* (33 bp). Fluorescently labeled annealed probes were analyzed by native PAGE (12% (w/v)), and RcnR/*rcnRA*Pro stoichiometry experiments were performed as described (50) by titration of RcnR (prepared in 100 mm NaCl, 400 mm KCl, 10 mm HEPES, pH 7.0, and 5 mm EDTA) into 2.5 μm *rcnRA*Pro in 60 mm NaCl, 240 mm KCl, 10 mm HEPES, pH 7.0, and 5 mm EDTA. For *K*_DNA_ determination in the absence of effector, *frmRA*Pro or *rcnRA*Pro was diluted to 10 nm in the same buffer. Formaldehyde, acetaldehyde, and GSH were included as outlined in the figure legends. For metal-loaded experiments, EDTA was replaced with 5 μm NiCl_2_ or CoCl_2_. FrmR (and variants) and RcnR (and variants) were prepared as described previously (50) and described above or by replacing EDTA with 1.2 molar eq/protein monomer of NiCl_2_ or CoCl_2_ as appropriate. Formaldehyde was prepared daily from single-use sealed ampules of methanol-free 16% (v/v) formaldehyde (Pierce) and stored under anaerobic conditions for the course of the experiment to prevent oxidation. A concentrated GSH stock (5 mm) was prepared in Chelex-treated, N_2_-purged 100 mm NaCl, 400 mm KCl, 10 mm HEPES, pH 7.0, under anaerobic conditions. This stock was confirmed to be ≥90% reduced by reaction with DTNB, stored anaerobically to prevent oxidation, and used within 3 days. Changes in anisotropy (Δ*r*_obs_) were measured using a modified Cary Eclipse fluorescence spectrophotometer (Agilent Technologies) as described (50). Control titrations of apo-FrmR and apo-FrmR^E64H^ into *frmRA*Pro (Fig. 4*C*) are new unpublished data sets and are presented here to demonstrate reproducibility and for comparative purposes. Data (for both FrmR and RcnR) were fit to a model describing binding of two non-dissociable tetramers (*K*_tet_ fixed at 10^−20^ m) to a target DNA probe with equal affinity (50, 86), using Dynafit (87) (see Figs. 3, 4, 6, and 7 legends and Table 1 for details; sample Dynafit script shown in the supplemental material). For experiments where DNA binding did not saturate, the average fitted Δ*r*_obs_ maximum value from apoprotein experiments was used as a fixed parameter. Coupling free energies (Δ*G*_C_) linking DNA binding to effector binding (62, 66, 67) were determined as described previously (50), calculated from the full set of (equally weighted) possible pairwise permutations of *K*_C_.

##### Crystallization of FrmR^E64H^ and Data Collection

Concentrated FrmR^E64H^ (∼1 mm) was diluted to 0.5 mm in 400 mm NaCl, 1 mm EDTA, 1 mm DTT, and 10 mm HEPES, pH 7.0, and stored aerobically at 4 °C for up to 2 weeks. Initial crystallization trials were conducted using the Screenmaker 96 + 8^TM^ Xtal (Innovadyne Technologies) and commercially available screening kits (Molecular Dimensions). Subsequent FrmR^E64H^ crystals were obtained in 20 mm NaCl, 23% (w/v) poly(ethylene glycol) 4000, and 10 mm BisTris, pH 6.5, by hanging drop vapor diffusion at 20 °C. Crystals were physically fragile and disintegrated rapidly when cryoprotectants were added. Multiple crystals using a wide range of cryoconditions were frozen and tested. Results were obtained from a crystal soaked in 25% (v/v) glycerol mounted in cryoloops (88). Overall data quality was compromised by residual ice rings and anisotropic diffraction, potentially giving rise to higher than expected *R*-factors of the final model. FrmR^E64H^ diffraction data were collected at the Diamond Light Source on beamline I03 at 77 K with a Pilatus pixel detector (89). Diffraction data were initially processed using Mosfilm (90) to a resolution of 2.1 Å to enable *ab initio* solution and reprocessed (to 2.19 Å) with Xia2 (91) for structure refinement. Initial molecular replacement trials using MolRep (92) and Phaser (93) using Protein Data Bank entry 2HH7 (Cu(I)-CsoR from *Mycobacterium tuberculosis*) (31) were unsuccessful, presumably due to differences in the orientation of the three helices of the monomer and significant differences in monomer-monomer as well as dimer-dimer orientations in the homotetramer. The structure was solved using Arcimboldo installed on a Condor grid computer (94, 95). The initial model was completed by iterative cycles of model building and refinement using COOT (96) and REFMAC (97). The final model contained one homotetramer in the asymmetric unit with each chain containing residues 2–89 and residue 90 in chains B and C and 103 water molecules. The structure was refined against intensities with local non-crystallographic symmetry restraints (98), using Phenix (99). Applying local non-crystallographic symmetry restraints enabled the tracing of all four chains despite relatively weak density, particularly in α3. This confers higher than expected overall real-space *R* value *Z*-score and *R*-factors. A number of polar surface residues where no electron density was observed for the side chains were refined as alanines (Chain A: His^3^, Lys^8^, Lys^9^, Glu^69^, Ile^82^; Chain B: Lys^62^, Glu^69^, Ile^82^, Leu^90^; Chain C: Glu^30^, Glu^69^, Leu^90^; Chain D: His^3^, Lys^8^, Lys^9^, Glu^55^, Lys^62^, Glu^69^, Ile^82^). Ramachandran plot analysis using RAMPAGE Ramachandran plot assessment (100) of FrmR^E64H^ demonstrates that 98.8% of residues are in the favored region (supplemental Fig. S1*B*). The final data and refinement statistics are provided in Table 2 with the structure deposited in the Protein Data Bank under accession code 5LCY.

**TABLE 2:** **Crystallographic data collection and refinement statistics for FrmR^E64H^**

Parameter	Value
**Data collection**	
Beam line	I03
Wavelength (Å)	0.9762
Space group	P2_1_
Cell dimensions	
*a*, *b*, *c* (Å)	68.79, 25.68, 100.50
α, β, γ (degrees)	90, 103.1, 90
Resolution (Å)	29.7–2.19
*R*_merge_	0.081 (0.495)*^a^*
*I*/σ*I*	12.1 (3.2)*^a^*
Multiplicity	6.3 (6.4)*^a^*
Completeness (%)	99.3
Wilson *B*-factor*^b^*	33

**Refinement**	
Resolution (Å)	29.7–2.19
No. reflections	17990
*R*_work/_*R*_free_	0.24/0.31
No. of atoms	
Protein	2767
Water	103
*B*-factors (Å^2^)	
Chain A	51
Chain B	47
Chain C	47
Chain D	53
Protein	30
Water	48
Root mean square deviations	
Bond lengths (Å)	0.01
Bond angles (degrees)	1.1

*^a^* The values in parentheses refer to the highest resolution shell (2.25–2.19 Å).

*^b^* Calculated using phenix.xtriage (99).

##### Protein Metal Migration by Size Exclusion Chromatography

Experiments were carried out as described previously (50). FrmR, FrmR^E64H^, FrmR^C35A^, or FrmR^H60L^ was incubated (120 min) with an excess of ZnCl_2_ in 100 mm NaCl, 400 mm KCl, 10 mm HEPES, pH 7.0, and an aliquot (0.5 ml) was resolved by size exclusion chromatography (PD10 Sephadex G25, GE Healthcare) in the same buffer conditions. Fractions were analyzed for zinc by inductively coupled plasma MS and for protein by a Bradford assay as described (50). The control experiments with FrmR and FrmR^E64H^ (Fig. 4*E*) are new unpublished data sets and are presented here to demonstrate reproducibility and for comparative purposes.

##### FrmR in Vivo Quantification and Detection of Met^1^ Cleavage by Liquid Chromatography-Tandem Mass Spectrometry

Quantification of FrmR in cellular lysates of SL1344 was performed exactly as described previously using aerobically grown logarithmic cells (50). To detect FrmR Met^1^ cleavage, a tryptic digest was performed with 5 μg of FrmR and 14 μg of trypsin in 50 mm NH_4_HCO_3_ with shaking (1000 rpm) at 37 °C for 16 h and stopped by the addition of 15% (v/v) formic acid (5 μl). The digested samples were separated by gradient elution at 0.3 ml min^−1^ using a Zorbax Eclipse Plus C18 column (2.1 × 150 mm, 3.5-μm particles, Agilent Technologies) at room temperature. Mobile phase A and B consisted of 0.1% (v/v) formic acid in water and 0.1% (v/v) formic acid in acetonitrile, respectively. Detection of FrmR amino-terminal peptide PHSPEDK was achieved by applying an aliquot (10 μl) to a 6500 triple quadrupole mass spectrometer (AB Sciex) operating in positive ionization mode. Acquisition methods used the following parameters: 5500 V ion spray voltage, 25 p.s.i. curtain gas, 60 p.s.i. source gas, 550 °C interface heating temperature, 40 V declustering potential, 26 V collision energy, and 27 V collision cell exit potential. Scheduled multiple-reaction monitoring of ion transition 405.19/488.24 was performed with a 90-s multiple-reaction monitoring detection window and 1.00-s target scan time.

##### Quantification of Intracellular Glutathione

Intracellular glutathione was measured as described (50). Lysates from logarithmically growing cells were prepared from overnight cultures grown in M9 minimal medium with glycerol as a carbon source, diluted 1:50 in fresh medium, and grown at 37 °C either in round bottom flasks with shaking to maintain aerobic conditions or with the addition of TMAO (40 mm) and static incubation of completely filled Parafilm-sealed 1.5-ml tubes to maintain anaerobic conditions. No growth was observed under anaerobic conditions when TMAO was not included as an electron acceptor. Viable cells were enumerated on LB agar, and cell volume was estimated as 1 fl.

##### Fractional Occupancy Model to Describe Formaldehyde Modification in Vivo

Fractional modification of FrmR, RcnR, and GSH with formaldehyde as a function of formaldehyde concentration was determined using Dynafit (87) with the following values as fixed parameters. Affinities of FrmR and RcnR for formaldehyde were estimated following fluorescence anisotropy to be 10^−5^ and 10^−4^ m, respectively; the dissociation constant for GSH and *S*-(hydroxymethyl)glutathione was 1.77 × 10^−3^ m (73); the total intracellular concentration of glutathione during anaerobic growth with TMAO as an electron acceptor was 1.2 × 10^−3^ m and was assumed to be in the reduced form (90–99% of the glutathione pool is GSH in resting *Salmonella* cells (101, 102)); the intracellular concentration of FrmR tetramer under aerobic conditions was calculated to be 1.61 × 10^−8^ m and was used as an estimate for the intracellular concentrations of FrmR and RcnR tetramers under anaerobic growth conditions. Cell volume was 1 fl. A sample Dynafit script is shown in the supplemental material.

## Author Contributions

D. O. carried out the *in vivo* survival and gene expression experiments and bioinformatic analysis and developed the fractional occupancy model for formaldehyde modification. C. P. did the *in vitro* metal-binding and fluorescence anisotropy experiments and generated FrmR^E64H^ crystals. E. P. and I. U. solved the x-ray crystal structure of FrmR^E64H^. J. C. and T. G. H performed the LC-MS/MS. L. N. S. and C. P. determined the intracellular glutathione concentration. D. O. and C. P. made equivalent contributions to data preparation and, with N. J. R., interpreted the significance of the data. D. O. and N. J. R. drafted the manuscript. N. J. R. had overall responsibility for the design and coordination of the program. All authors reviewed the results and edited and approved the final version of the manuscript.

## Supplementary Material

Supplemental Data

## Footnotes

3 The abbreviations used are:

TMAO: trimethylamine *N*-oxide
MNIC: maximum non-inhibitory concentrations
BisTris: 2-[bis(2-hydroxyethyl)amino]-2-(hydroxymethyl)propane-1,3-diol.
