# Supplementary material for: The Effectors and Sensory Sites of Formaldehyde-responsive Regulator FrmR and Metal-sensing Variant
Source: J Biol Chem. 2016 Jul 29;291(37):19502–16. doi: 10.1074/jbc.M116.745174 (PMC5016687; doi:10.1074/jbc.M116.745174)
Supplement: Supplemental Data [file supp_291_37_19502__index.html]

The Effectors and Sensory Sites of Formaldehyde-Responsive Regulator FrmR and Metal-Sensing Variant — The Effectors and Sensory Sites of Formaldehyde-responsive Regulator FrmR and Metal-sensing Variant — The Effectors of FrmR — Supplemental Data 

# The Effectors and Sensory Sites of Formaldehyde-responsive Regulator FrmR and Metal-sensing Variant

## Supplemental Data

- Supplemental Data (.pdf, 475 KB) - Supplemental figure S1, figure S2, table S1, table S2 and sample Dynafit scripts.
