# Supplementary material for: The Effectors and Sensory Sites of Formaldehyde-responsive Regulator FrmR and Metal-sensing Variant
Source: J Biol Chem. 2016 Jul 29;291(37):19502–16. doi: 10.1074/jbc.M116.745174 (PMC5016687; doi:10.1074/jbc.M116.745174)
Supplement: Supplemental Data [file 10.1074_M116.745174_jbc.M116.745174-1.pdf]

Supplemental Data

The Effectors and Sensory Sites of Formaldehyde-Responsive Regulator FrmR and Metal-Sensing Variant

Deenah Osman[‡1], Cecilia Piergentili[‡1], Junjun Chen[§], Lucy N. Sayer[‡], Isabel Usón[¶‖], Thomas G. Huggins[§], Nigel J. Robinson[‡] and Ehmke Pohl[‡]

From the [‡]Durham University, School of Biological and Biomedical Sciences, Department of Chemistry, DH1 3LE, [§]Procter and Gamble, Mason Business Centre, Cincinnati, Ohio 45040, USA, [¶]Instituto de Biología Molecular de Barcelona (IBMB-CSIC) Barcelona Science Park, Barcelona, Spain, and [‖]ICREA , Pg. Lluís Companys 23, 08010 Barcelona, Spain.

CONTENTS

FIGURE S1	S-2
FIGURE S2	S-3
TABLE S1	S-4
TABLE S2	S-5
DYNAFIT SCRIPTS	S-6

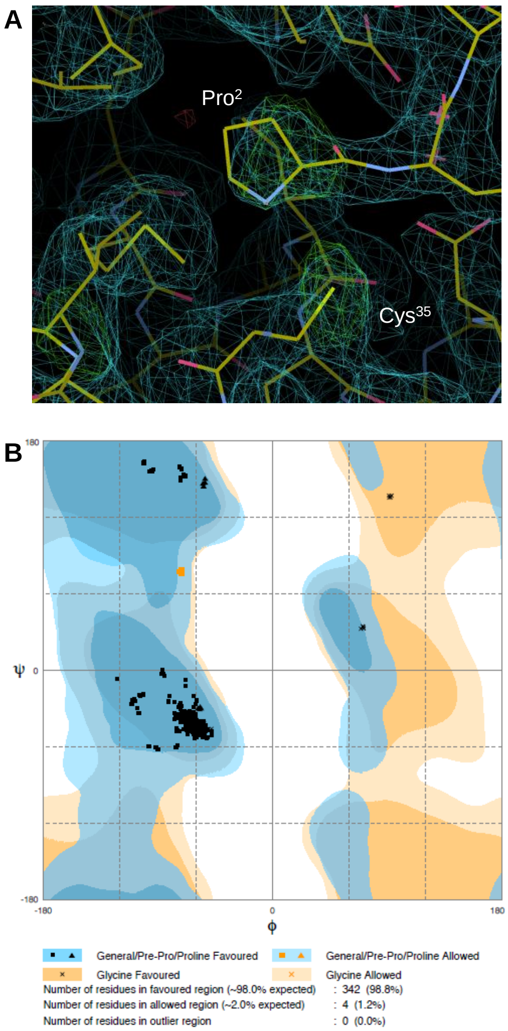

FIGURE S1. Omit map and Ramachandran plot analysis for the FrmR^{E64H} crystal structure. *A*, Representative omit map of the N-terminal Pro^{2} (chain B) and Cys^{35} (chain A) of FrmR^{E64H}. Pro^{2} and the sulfur atom of Cys^{35} were excluded from the model prior to (ten rounds of) refinement and consecutive calculation of phases, in order to minimize model bias. The 2Fo-Fc omit map (1σ, blue) and Fo-Fc omit map (3σ, green) unambiguously confirm the position of Pro^{2} and Cys^{35} side chain. The maps are non-averaged by the four-fold non-crystallographic symmetry. *B*, Ramachandran plot analysis of FrmR^{E64H} using RAMPAGE Ramachandran Plot Assessment (100). The general and Pro-Pro favored regions are shown in dark blue; the general and Pro-Pro allowed regions are shown in pale blue; the glycine favored and allowed regions are shown in dark and pale orange, respectively; the disallowed region is in white. The plot demonstrates that 98.8 % of residues are in the favored region.

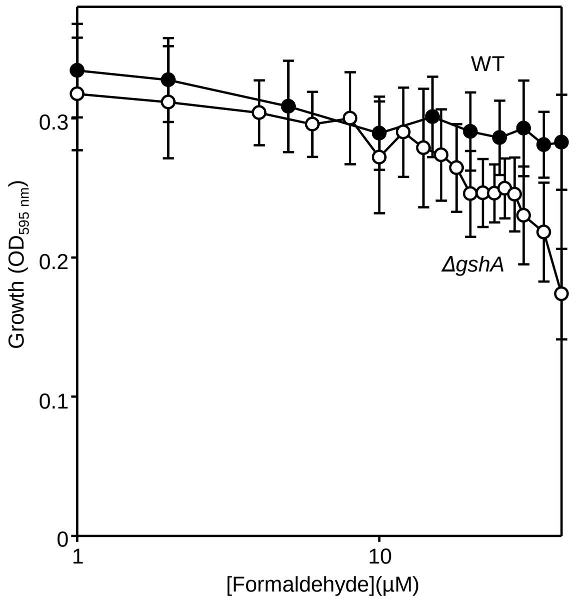

FIGURE S2. Growth of SL1344 and *ΔgshA* containing P*frmRA*-*frmR*.
Growth of SL1344 (*solid symbols*) and *ΔgshA* (*open symbols*) containing P*frmRA*-*frmR* fused to *lacZ* grown to mid-exponential phase in M9 minimal medium in the presence of formaldehyde. Data correspond to cultures used in Figure *8B*.

TABLE S1. **Oligonucleotides used in this study**

No.	Primer name	Sequence
**1**	PfrmRA-frmRP2S_F	5′-GTATATGGAGGTCGAATGTCGCATTCACCTGAAG-3′
**2**	PfrmRA-frmRP2S_R	5′-CTTCAGGTGAATGCGACATTCGACCTCCATATAC-3′
**3**	PfrmRA-frmRC35A_F	5′-GAGTCTGGCGAACCTGCTCTGGCGATTCTGCAAC-3′
**4**	PfrmRA-frmRC35A_R	5′-GTTGCAGAATCGCCAGAGCAGGTTCGCCAGACTC-3′
**5**	rcnRS2P-PrcnRA_F	5′-AGTGAGGTGTTGTAATGCCACATACCATCCGGGAC-3′
**6**	rcnRS2P-PrcnRA_R	5′-GTCCCGGATGGTATGTGGCATTACAACACCTCACT-3′
**7**	pETfrmR_P2S_F	5′-GGAGATATACATATGTCGCATTCACCTGAAGATAAAAAACGTATCC-3′
**8**	pETfrmR_P2S_R	5′-GGATACGTTTTTTATCTTCAGGTGAATGCGACATATGTATATCTCC-3′
**9**	pETfrmR_C35A_F	5′-GAGTCTGGCGAACCTGCTCTGGCGATTCTGCAAC-3′
**10**	pETfrmR_C35A_R	5′-GTTGCAGAATCGCCAGAGCAGGTTCGCCAGACTC-3′
**11**	pETfrmR_H60L_F	5′-GTGAAATGGTTGAAATCCTTCTGAAAGATGAGCTGGTCAG-3′
**12**	pETfrmR_H60L_R	5′-CTGACCAGCTCATCTTTCAGAAGGATTTCAACCATTTCAC-3′
**13**	pETrcnR_S2P_F	5′-GAAGGAGATATACATATGCCACATACCATCCGGGACAAAC-3′
**14**	pETrcnR_S2P_R	5′-GTTTGTCCCGGATGGTATGTGGCATATGTATATCTCCTTC-3′
**15**	rcnRAPro_F[a]	5′-[HEX]TACTCCCCCCCAGTATAGAATACTACCCCCCAGTA-3′
**16**	rcnRAPro_R[a]	5′-TACTGGGGGGTAGTATTCTATACTGGGGGGGAGTA-3′
**17**	frmRAPro*_F	5′-[HEX]TTCTGGTTCAACACCCCCCTATAGTATATGGAG-3′
**18**	frmRAPro*_R	5′-CTCCATATACTATAGGGGGGTGTTGAACCAGAA-3′

[a]Features of the *Salmonella* RcnR-binding site: G/C tracts are underlined. T/A-rich inverted repeats are highlighted in grey.

.

TABLE S2. **MRM LC/MS data for FrmR quantitation in cell lysates**
NA means not applicable. STD 1-6 are known concentrations of FrmR which make up a front and back standard curve. LQC is low quality control. HQC is high quality control. SL1344 1-3 are independent cell lysate preparations of *Salmonella* SL1344 (n=3).

**Sample[a]**	**Actual [FrmR] (ng/ 100 µl)**	**Analyte Peak Height[c] (counts s^{-1})**	**Internal Standard Peak Height[d] (counts s^{-1})**	**Analyte/ Internal Standard**	**Calculated [FrmR][b] (ng/ 100 µl)**	**Calculated [FrmR]/ Actual [FrmR]**
**STD 1**	5	2.38E+04	2.95E+05	0.08	4.96	0.99
**STD 2**	10	4.62E+04	2.86E+05	0.16	10	1.00
**STD 3**	50	2.15E+05	2.74E+05	0.78	48.5	0.97
**STD 4**	250	1.26E+06	2.90E+05	4.34	254	1.02
**STD 5**	425	2.11E+06	2.90E+05	7.28	406	0.96
**STD 6**	500	2.63E+06	2.79E+05	9.43	511	1.02
**LQC**	12	5.78E+04	2.72E+05	0.21	13.2	1.10
**HQC**	400	2.22E+06	2.73E+05	8.13	448	1.12
**SL1344 1**	NA	3.34E+04	2.12E+05	0.16	9.79	NA
**SL1344 2**	NA	2.90E+04	2.45E+05	0.12	7.33	NA
**SL1344 3**	NA	3.33E+04	2.44E+05	0.14	8.46	NA
**LQC**	12	4.70E+04	2.11E+05	0.22	13.9	1.16
**HQC**	400	1.77E+06	2.16E+05	8.19	451	1.13
**STD 1**	5	1.59E+04	1.93E+05	0.08	5.06	1.01
**STD 2**	10	3.13E+04	1.98E+05	0.16	9.83	0.98
**STD 3**	50	1.63E+05	1.91E+05	0.85	52.8	1.06
**STD 4**	250	8.37E+05	2.01E+05	4.16	244	0.98
**STD 5**	425	1.48E+06	1.98E+05	7.47	416	0.98
**STD 6**	500	1.79E+06	1.88E+05	9.52	516	1.03

[a]Samples were analysed in the order listed. [b]Calculated using the two standard curves shown using a quadratic $1/x^2$ weighted regression model. [c]Analyte peptide is GQVEALER. [d]Internal standard is labelled GQVEALER[$^{13}C_6$, $^{15}N_4$].

DYNAFIT SCRIPTS.

1. **Script to describe the binding of apo-RcnR or apo-FrmR to *rcnRA*Pro or *frmRA*Pro, respectively**

```
[model]
     Two RcnR (or FrmR) tetramers bind rcnRAPro (or frmRAPro) with
     equal affinity

[components]
     ; P        =    protein monomer
     ; D        =    double-stranded DNA probe

[task]
     task       =    fit
     data       =    equilibria

[mechanism]
     P + P + P + P   <==> P4          :  Keq1   dissociation
     P4 + D          <==> (P4)D       :  Keq2   dissociation
     P4 + (P4)D      <==> (P4)2D      :  Keq2   dissociation

[concentrations];nanomolar
     D = 10

[constants]          ;nanomolar
     Keq1       =    0.00000000001
     Keq2       =    estimate based on titration ?

[responses]
      (P4)D     =    0.5 *(final r - initial r)/[D]
     (P4)2D     =    (final r - initial r)/[D]    ?

[data]
     directory       C:/data
     variable        P
     offset     =    auto
     file            example.txt

[output]
     directory       C:/output

[end]

r = anisotropy

Parameters highlighted in grey were replaced with:
Keq2 = 200 (RcnR) and 100 (FrmR)
(final r - initial r) = 0.09629 (RcnR) and 0.03458 (FrmR)
```

DYNAFIT SCRIPTS continued.

2. **Script to describe the fractional modification of FrmR by formaldehyde under anaerobic growth conditions**

```
[components]
     ; FrmR      =     FrmR tetramer
     ; GSH       =     reduced glutathione
     ; F         =     formaldehyde

[task]
     task       =     fit
     data       =     equilibria

[mechanism]
     FrmR + F         <==> FrmR.F      :    Keq1   dissociation
     GSH  + F         <==> GSH.F       :    Keq2   dissociation

[constants]           ; molar
     Keq1 = 0.00001
     Keq2 = 0.00177

[concentrations]      ; molar
     FrmR = 1.61E-8
     GSH  = 0.0012

[responses]           ; FrmR.F = 1/[FrmR]
     FrmR.F = 6.21E+7

[data]
     directory        C:/data
     variable         F
     offset     =     auto
     file             example.txt

[output]

     directory        C:/output

[end]
```
